# Making it on the breadline – improving food security on the Anangu Pitjantjatjara Yankunytjatjara Lands, Central Australia

**DOI:** 10.1186/s12889-024-20495-9

**Published:** 2024-11-08

**Authors:** Amanda J. Lee, Stephan Rainow, Liza Balmer, Rhiannon Hutchinson, Suzanne Bryce, Meron Lewis, Lisa-Maree Herron, Paul Torzillo, Robert Stevens, Margaret Kavanagh, Lisa Wells, Ingrid Kenny, Nganampa Health Council, Stephan Rainow, Paul Torzillo, Jamie Nyaningu, John Singer, Sue Haines, Ngaanyatjarra Pitjantjatjara Yankunytjatjara Women’s Council, Liza Balmer, Rhiannon Hutchinson, Suzanne Bryce, Margaret Kavanagh, Lisa Wells, Ingrid Kenny, Kunmanara Smith, Yangi Yangi Yangi Yangi Fox, Maureen Baker, Yanyi Bandicha, Janet Forbes, Rene Kulitja, Wanatjura Lewis, Peggy Naylon, Tjawina Nellie Roberts, Lily Tjiweri, Martha Ward, Carmen Windy, Lynette Ross

**Affiliations:** 1 School of Public Health, The University of Queensland, Herston, QLD 4006 Australia; 2 Nganampa Health Council, Alice Springs, Northern Territory, 0870 Australia; 3 Ngaanyatjarra Pitjantjatjara Yankunytjatjara Women’s Council, Alice Springs, Northern Territory, 0870 Australia; 4 The University of Sydney, Newtown, NSW 2042 Australia; 5 Mai Wiru Regional Stores Aboriginal Corporation, Alice Springs, Northern Territory, 0870 Australia

**Keywords:** First Nations, Food security, Retail nutrition policy, Store monitoring and surveillance, Non-communicable disease, Longitudinal case study, Co-design, Community control

## Abstract

**Background:**

This longitudinal case study describes the efforts and impacts of community-controlled service organisations on the Anangu Pitjantjatjara Yankunytjatjara (APY) Lands in Central Australia to tackle food security since the 1980s, with a focus on the last decade, particularly during a year of concerted action from mid-2018.

**Methods:**

The co-designed study comprised an interrupted time series with controls. Availability, affordability, accessibility and sales of foods in the community retail stores on the APY Lands were monitored regularly from 2014 to mid-2022, including by local research teams. Store nutrition policy was updated early 2018. For a year from mid-2018, of the eight communities with stores: (i) two were the focus for concerted intervention, including support from a locally based project officer to help implement the policy and action 105 community requests for nutrition activities (ii) three received usual support to implement the policy; and (iii) three were subject to ‘business as usual’. From mid-2019, all communities/stores received usual service, from 2020 with some restrictions related to the COVID-19 pandemic. Results were compared over time, across different community/store groups and with controls.

**Results:**

In the 12 months from mid-2018, all food security metrics improved most in the two focus communities. Impacts were less marked in the communities without additional support to implement the revised nutrition policy, and even less apparent, although more varied, in the other three communities/stores. Dietary intake improved only in the two focus communities.

In all communities from early 2020 most gains eroded due to impacts of the COVID-19 pandemic and other external stressors. Food security metrics, including price of healthy food, appeared more resilient in the focus communities, although diet quality worsened. At all times assessed, healthy diets were unaffordable for welfare-dependant households.

**Conclusions:**

This co-designed study demonstrates the effectiveness of community-led approaches, confirming that it is possible to improve food security and diet in remote Aboriginal communities. However, sustained action and monitoring, dedicated resources and employment of local people are critical for success. Results also highlight that low incomes are a major barrier to food security.

**Supplementary Information:**

The online version contains supplementary material available at 10.1186/s12889-024-20495-9.

## Background

Since colonisation, First Nations Peoples in Australia, Aboriginal and Torres Strait Islanders, have experienced poorer health than non-Indigenous people, with higher rates of premature death and a life expectancy at least eight years less [1]. More than three-quarters of these premature deaths are from preventable non-communicable diseases (NCDs), such as cardiovascular disease, diabetes, renal disease and some cancers [2]. Many major risk factors that contribute to these conditions are related to poor diet, characterised by inadequate intake of fruit, vegetables and other nutritious foods, and overconsumption of unhealthy food and drinks [2–5]. The most recently conducted National Nutrition and Physical Activity Survey (2011–13) found that unhealthy (‘discretionary’ and/or ‘ultra-processed’) foods and drinks contribute 35% of dietary energy intake of Australian adults, and 41% of intake of Aboriginal and Torres Strait Islander adults [3]. Such a diet contrasts dramatically with the varied, healthy, traditional diets consumed by First Nations groups prior to colonisation [5].

Nearly one-fifth of the Aboriginal and Torres Strait Islander population in Australia reside in remote communities. These communities bear a disproportionate burden of preventable NCDs, which account for 40% of the health gap between Indigenous and other Australians [6–8]. In these areas particularly, poor diet is a result of lack of food security, which exists when “all people, at all times, have physical, social and economic access to sufficient, safe and nutritious food that meets their dietary needs and food preferences for an active and healthy life” [9]. Food security is underscored by the availability, affordability, accessibility and acceptability of healthy foods [10, 11].

These dietary factors are determined by complex historical, socioeconomic, environmental, ecological, cultural and political factors, including intergenerational trauma and disruption to family structures, lower incomes, lower educational opportunities and attainment; higher rates of unemployment; poorer access to a healthy and affordable food supply; poorer access to health infrastructure including adequate housing and food preparation and storage facilities; over-crowding, poor transport, high food costs, and lack of Indigenous food sovereignty [4, 5, 11, 12].

As a proxy for assessment of food insecurity, the National Australian Aboriginal and Torres Strait Islander Health Survey (NATSIHS) in 2012–13 and 2018–19 found that more than one in five (22% and 26% respectively) Aboriginal and Torres Strait Islander people reported living in a household that, in the previous 12 months, had run out of food and could not afford to buy more [3, 8]. This was much higher than available data in the non-Indigenous population (3.7% in 2011–12) [13]. In 2012–13, Aboriginal and Torres Strait Islander people living in remote areas were more likely to run out of food than those in non-remote areas (31% and 20% respectively) [3]; these figures had worsened by 2018–19, although comparisons are difficult due to differences in reporting categories [12].

Focus on such challenges has been criticised as taking a deficit approach by failing to recognise the strengths and resiliency of First Nations Peoples in Australia [14, 15]. For example, these data also highlight that more than two thirds of Aboriginal and Torres Strait Islander people in remote areas who had run out of food and couldn’t afford to buy more *were* able to source something for their families to eat [3], likely achieved through cultural knowledge and relationship networks. Also, it has been demonstrated previously that rapid, sustained improvements in objective measures of food security, dietary intake, nutrition status, anthropometric and diet-related biomedical risk factors for NCD *are* possible [16–18]. Further, studies show consistently that co-design and strong Aboriginal and Torres Strait Islander community ownership/leadership is an essential element of effective nutrition interventions [5, 19–22]. The evidence base demonstrates clearly that community-led dietary interventions and supportive policy settings, with an ‘upstream’ focus on both food supply and demand, have enormous potential to reduce health inequalities between Aboriginal and Torres Strait Islander groups and non-Indigenous Australians [5, 19, 20, 23].

Despite this, nutrition and food security have been largely absent from formal national strategies to improve Aboriginal and Torres Strait Islander health in Australia [24, 25], with analysis suggesting this is due to perceived ‘complexity’ of the problem, among other challenges [5, 26]. However, there have been several national inquiries, most recently the House of Representatives Standing Committee on Indigenous Affairs Inquiry into food pricing and food security in remote Indigenous communities [11]. While over 100 submissions were received, showing no shortage of suggestions, very few reported implementations of community-led solutions, such as those described in this paper.

**Fig. 1:**
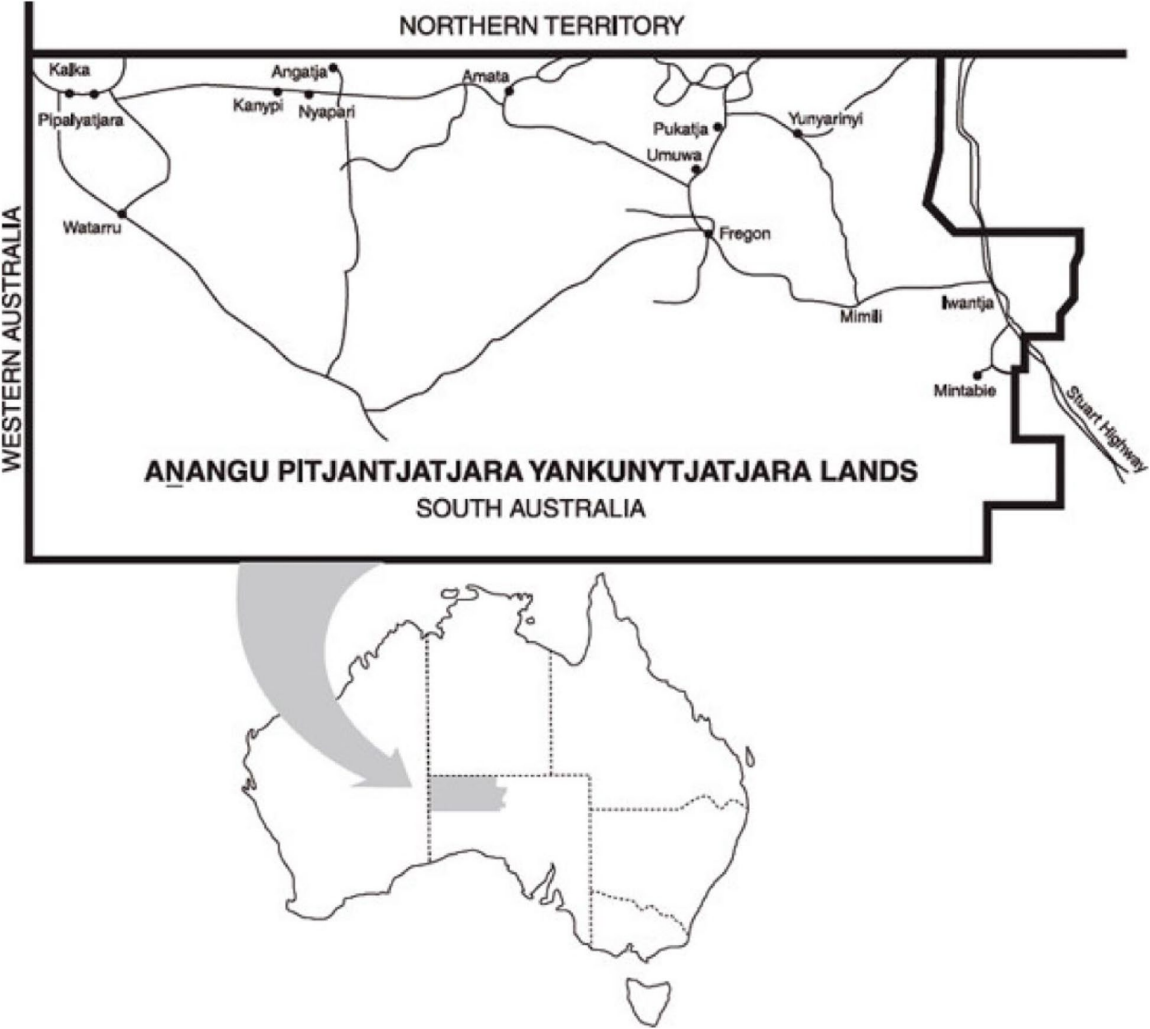
Map showing the Anangu Pitjantjatjara Yankunytjatjara (APY) Lands

This study is centred on the Anangu Pitjantjatjara Yankunytjatjara (APY) Lands, a remote area of 102,650 km^2^ in Central Australia. The Lands are home to more than 3000 Anangu, the Aboriginal traditional owners who hold inalienable freehold title under the Pitjantjatjara Land Rights Act 1981 [27] (Fig. 1). Anangu live in seven communities and more than 40 homelands in the area, and maintain strong connections to their land, culture, language and history. They have a strong voice on all issues affecting their lives, including health and wellbeing, which is facilitated by traditional structures and local, community-controlled service organisations. Among these, Nganampa Health Council is an Aboriginal owned and controlled health service established in 1983, which operates primary care and public health programs on the APY Lands [28]. The Ngaanyatjarra Pitjantjatjara Yankunytjatjara Women’s Council (NPY Women’s Council), established in 1980, is an Aboriginal organisation that provides advocacy and support services to Anangu women and their families [29]. The Mai Wiru^1^ Regional Stores Council Aboriginal Corporation (Mai Wiru) [30] was instigated following a cost of living study commissioned by Anangu Pitjantjatjara Services in 1998 [31]. This study found that Anangu families experienced “hungry days” up to three days a week, surviving on sugary tea and damper (a type of bread made from flour, water and baking powder cooked on an open fire) or *arngu* (flour gruel) because they could not afford to buy adequate, healthy food [31].

During the years of this extended case study, five of the seven larger retail stores on the APY Lands were managed by Mai Wiru [30], one by Outback Stores [32], and the other by the local community council, with three smaller ‘convenience’ stores managed by private operators. There was only one store in each of the larger communities on the APY Lands, apart from one community that was serviced by both a Mai Wiru store and a large, privately-run convenience store. All communities in the study are located 415 to 716 km by road from the closest regional town centre, Alice Springs in the Northern Territory. Population in each community with a retail store is highly variable, ranging from around 60 in the smallest places to over 400 in the two largest centres; mobility between communities and Alice Springs is usually high.

This study built on efforts to improve food security and diet on the APY Lands dating from the early 1980s, published previously [33]. Improving nutrition is a key focus of Nganampa Health Council’s clinical services, especially in maternal and infant health and management of NCDs. Wider food security work sits under Nganampa Health Council’s Uwankara Palyanyku Kanyintjaku (UPK) program [34] that collaborates with Mai Wiru to improve food supply. Family-focused support for children with growth faltering and/or other diet-related health problems, and broader community nutrition education initiatives are provided by the Child Nutrition Program of the NPY Women’s Council [29]. In 2002, the first Mai Wiru Regional Stores Policy and a Stores Nutrition Handbook were developed by Nganampa Health Council and the NPY Women’s Council and supported by the overarching Anangu Pitjantjatjara Yankunytjatjara (APY) Council [27] and all Aboriginal Communities on the APY Lands [30].

Implementation delivered mixed results [33]. For example, in Mai Wiru stores on the APY Lands from 1986 to 2012, fruit and vegetable intake doubled, and the proportion of energy intake derived from sugar decreased from 30 to 22%. However, the intake of sugar sweetened beverages (SSBs) increased four-fold and the proportion of energy derived from unhealthy foods, especially take-away foods, increased markedly [33]. Several components of the Mai Wiru Regional Stores Policy were no longer being implemented. Those store managers who were aware of the nutrition policy considered it to be “out-of-date”. During this time, the demand for clinical services to treat diet-related disease continued to escalate on the APY Lands [35].

All available store data from the APY Lands reflected broader changes to the general Australian food supply. This reinforced the notion that, in the absence of national market regulation, concerted implementation of promising targeted intervention strategies – not only by the store, but also by schools, clinics and the wider community – were likely to be required to improve food security and diet and prevent and manage NCDs on the APY Lands [33].

While most previous studies focused on the ‘average’ community diet, in 2016 an ethnographic study by the NPY Women’s Council [36] identified three main dietary patterns at household level, and a wide range of determinants influencing food practices and choices, including economic cycles, structure, organisation and mobility; housing and available resources including for food storage, preparation and cooking (e.g. only 8% of houses had functional kitchens); and familiarity and convenience. Depending on the level of food stress and the number of mouths to feed (especially the number of children), household dietary patterns were dominated by either unhealthy takeaway foods or cereal (grain) foods such as bread, damper and *arngu*. Importantly, the study highlighted the resourcefulness and resiliency of Anangu, who managed to secure food for their families despite poverty and adversity [36].

Drawing on these insights, there were renewed, concerted efforts to improve food security and diet on the APY Lands. This study details the implementation and ongoing impacts of these community-led endeavors, including in an intensive year of action from mid-2018 to mid-2019, and during the COVID-19 pandemic from early 2020 until mid-2022.

### Aim

The aim of this study was to describe implementation, impacts and outcomes of community-led efforts to improve food security and diet in remote Aboriginal communities in Central Australia during the past decade, particularly during a year of concerted action from mid-2018, and during the subsequent COVID-19 pandemic.

## Methods

### Study design

**Fig. 2:**
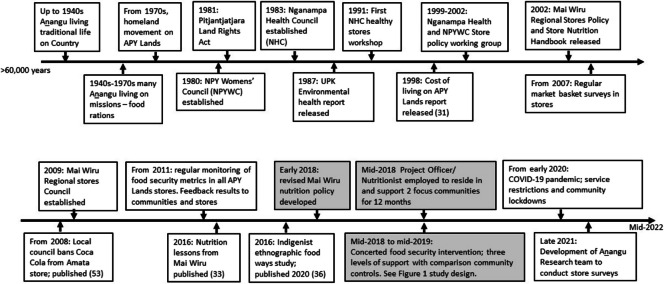
Timeline of selected events and actions mentioned in the text

The study was co-designed iteratively by representatives from the NPY Women’s Council, Nganampa Health Council, Mai Wiru, key community members, and invited research collaborators (the Steering Committee). The broad case study incorporated an interrupted time-series design with a concerted, community-led intervention to improve food security focused on two communities with Mai Wiru retail stores from mid-2018 to mid-2019, and three groups of control/comparator communities for available metrics. A high-level Advisory Committee of respected national Aboriginal nutrition and health experts met twice to provide recommendations to strengthen the study design and approach. Table 1 summarises the study design including the approach and the roles of all participating communities/stores (Table 1) and timeline (Fig. 2).

**Table 1:** Study design 1A: Key features

**Aim**	**Objectives**	**Strategies**	**Process Evaluation** **(Assessment of whether strategies were implemented as intended)**	**Impact evaluation** **(Assessment of whether objectives were achieved)**	**Outcome evaluation** **(Assessment of whether aims were achieved)**
To improve food security and diet in the commun-ities on the APY Lands	To: (i) improve food supply (availability, affordability, placement, promotion) and (ii) increase demand for healthy food In all stores on the APY Lands (*n*=8)	**Business as usual 2014-mid-2022 - all stores/times with exception of below: ** (i and ii) Monitoring and surveillance of food availability, placement and promotion in all stores on the APY Lands (*n*=8); feedback results to store groups/communities Monitoring of food price all stores, including convenience store outside APY Lands and supermarkets in Alice Springs **2018-2019:** (i) early 2018 revision of updated nutrition policy in 5 Mai Wiru community stores, with supported implementation from mid-2018 to mid-2019 by a specifically employed project officer in two Mai Wiru stores in the intervention focus communities (ii) community-led nutrition promotion/ food literacy/food sovereignty/budgeting activities from mid-2018 to mid-2019 by the project officer in the two intervention focus communities	**All stores, all times** Quantity, reach, quality of monitoring, surveillance, reporting and feedback strategies **Mid-2018 to mid-2019** (i) Quantity, reach, quality of store nutrition support strategies provided to the two intervention focus communities (ii) Quantity, reach, quality of nutrition support activities provided to the two intervention focus communities	**All stores, all times** Assessment of food availability, affordability, accessibility, acceptability, advertising/promotion (regular store audits against revised store nutrition policy) Comparison of results in groups of communities (by three intervention levels)	**All stores, all times** Assessment of community diet (validated store-turnover: pre and post study where available) Comparison of results in intervention focus and other groups of communities (by three intervention levels) Relationship between process, impact and outcome measures

Community/store codes:

• Participating communities/stores (*n*=12) on APY Lands (*n*=8) and in control communities/ outside APY Lands (*n*=4), comprising:

• Intervention focus communities with Mai Wiru stores (*n*=2). Codes: IMW1 and IMW2

• Nutrition Policy Control communities/stores with Mai Wiru stores (*n*=3) Codes: PCMW3, PCMW4, PCMW5 (business as usual, with introduction of revised Mai Wiru store nutrition policy early in 2018).

• Other Control communities/stores on APY Lands with non-Mai Wiru stores (*n*=3) Codes: CAPY6, CAPY7, CAPY8 (business as usual)

• Comparison Convenience Store located close to, but outside, the APY Lands (*n*=1) Codes CS9 (price data only)

• Regional centre Comparison supermarkets in Alice Springs (*n*=2) Codes: CAS10, CAS11 (price data only). At community request from January 2019, a supermarket popular with Aboriginal customers (CAS12) was added to the survey (total *n*=3)

### Participating communities and stores

Community and retail store participation in the study was voluntary. Consistent with the requirements of ethical approval, individual communities and stores are not identified; codes are used to ensure anonymity; details are provided in Table 1. The Steering group recommended that two communities with Mai Wiru stores that had been requesting additional nutrition support for some years be invited to participate as the focus sites for implementation. The relevant community councils and store committees were approached and agreed to participate as outlined in Table 1. Permission for the specifically appointed project officer/nutritionist and broader research team to work and consult with community members was also sought and gained from Elders and representatives of all relevant community groups and services (including child play groups, schools, and aged care facilities). Permission for the project officer, community members and broader research team to work with retail store staff to help implement the revised Mai Wiru nutrition policy was sought formally and gained from the local store committees at the Intervention focus stores- IMW2 in March 2018 and later at IMW1. The other three communities with Mai Wiru stores on the APY Lands (PCMW3, PCMW4 and PCMW5) participated as nutrition policy controls; these community stores were managed by Mai Wiru, and so were subject to the revised store policy, but did not receive additional assistance with policy implementation, and community requests for nutrition promotion activities in those communities were met by usual service provision. The three other communities on the APY Lands with stores managed under different models (independently or by Outback Stores, in no specific order—CAPYS6, CAPYS7, CAPY8) were subject to ‘business as usual’ for both objectives. Food prices only were also collected from a convenience store outside but close to the APY Lands (CS9) and three supermarkets in Alice Springs (CA10, CA11 and CA13). By 2020, several smaller convenience stores that had been operating on the APY Lands had closed so they were not included in the study.

The project followed the principles of co-design [37, 38], community Participatory Action Research [39] and the knowledge-to-action ethics framework developed by the Canadian Institutes of Health Research [40], and these principles were embedded in service delivery. Specific strategies informed by Anangu Knowledges of cultural food ways (see below) [36] were developed and refined iteratively by the steering group, with input from participating organisations, key community members and the advisory committee. These were fine-tuned by collective consideration of the results of the regular audits of store and community activities during the concerted intervention which ran from mid-2018 to mid-2019. Where and when possible, relevant data were collected from 2014 to 2022 inclusive.

### Anangu knowledges and definition of healthy foods

Anangu classify traditional foods as *kuka* (animal foods, such as kangaroo and emu), *mai* (plant foods, including seeds, nuts, fruit, tubers, leaves), *maku* (edible grubs) and *tjuratja* (sweet foods, such as flowers and lerp) [41]. Building on strong community knowledge of traditional foods and holistic concepts of health, healthy food and drinks were defined consistent with the two Australian Dietary Guidelines (ADGs) most relevant for Aboriginal and Torres Strait Islanders [4], which are:

- enjoy traditional foods whenever possible, and
- when choosing store foods, select those most like traditional bush foods, such as fresh plant foods, wholegrain (cereal) foods, seafoods, and lean meats and poultry.

Therefore, heathy foods and drinks are reported in terms of groups that are most like traditional foods. ‘Discretionary’ and/or ‘ultra-processed’ foods and drinks introduced by non-Indigenous colonisers are reported as unhealthy foods. In particular, Anangu noted “sugar *kura*” (sugar is ‘bad’) and associated SSBs especially with many of the diet-related health issues suffered on the APY Lands. In this paper, drinks are usually included as “food”.

### Strategies to improve food supply 2018–2019

The Mai Wiru Store Nutrition Policy was revised early in 2018 with the input of Mai Wiru staff, local store committees, representatives of Nganampa Health Council and the NPY Women’s Council, and a registered dietitian (KE) with over 20 years’ experience working in nutrition policy in remote community stores. Policy content revised or updated included standards for takeaway outlets, foods suitable for infants and those with chronic diseases, fresh produce sections in the stores, store ordering practices, and in-store price cross-subsidisation. After consultation on three iterative drafts, all members of the steering committee approved the final version. Mai Wiru informed all their store managers and store committees of the revised nutrition policy requirements, with the expectation that all components of the policy would be implemented in each of the five Mai Wiru stores on the APY Lands.

In the two focus communities (IMW1 and IMW2) community members and the community-based project officer/nutritionist (RH) worked with store managers, retail store committees and staff to support implementation of the revised Mai Wiru nutrition policy. Examples of support activities included development of colourful and attractive displays of healthy foods and drinks, such as fresh produce, at checkout counters and ends of shelves at the front of the stores, and conducting point-of-sale promotions, such as cooking demonstrations and visual display of sugar content of SSBs.

The availability, placement and promotion of healthy and unhealthy products was monitored regularly in all stores on the APY Lands and the results were reported back to store committees, store managers, service providers and communities to inform future endeavours.

### Strategies to increase demand for healthy food and drinks

Community-led nutrition promotion activities commenced in both IMW1 and IMW2 by August 2018. Consistent with NPY Women’s Council’s *malparara*^2^ philosophy, community members were employed to work alongside the project officer, ensuring all activities were culturally relevant and built on local Knowledges. In collaboration with community organisations, such as schools, youth groups, playgroups, Home and Aged Care (HAAC) services, and the Regional Anangu Services Aboriginal Corporation (RASAC) Community Development Programme (CDP) all community requests to help increase demand for healthy food and drinks were facilitated by the project team. Their remit was “to do whatever community members asked, to help improve nutrition”. Activities included supporting traditional practices (such as bush excursions, including ‘bush picnics’ and traditional bush food gathering); practical cooking workshops; development of healthy food budgeting resources and posters promoting quick, easy, inexpensive recipes; and conducting food-based activities with community services and programs such as after school and aged-care programs. The project officer maintained a detailed activity diary in Google documents, recording all approaches for help, resources or support from community members and store staff. All activities conducted were documented, classified and enumerated to inform process evaluation.

### Store nutrition benchmarking, monitoring, surveillance and iterative co-design

Evaluation was built into the study from inception. Where available, food price, affordability, availability, placement in the store and promotion data were collected from all participating stores at least yearly from 2014 to 2022, with the exception of 2020 due to travel restrictions related to the COVID-19 pandemic. The results of each survey were analysed by the team at the University of Queensland, reported to the members of the steering group and shared with participating store managers, store committees, community leaders/elders, community groups, *malpas* and other key community members for discussion and advice, to inform iterative decisions around subsequent steps. Examples of the store survey reports can be seen on the NPY Women’s Council, Nganampa Health and the Australian Prevention Partnership Centre websites [28, 29, 42].

### Impact evaluation and tools

The impacts of the study were evaluated by change in food availability, product placement and promotion, food price, diet cost and affordability over time. Metrics were assessed using the survey instruments and tools described below and included in Supplementary File 1. The Food Index for Remote Stores (FIRST) tool was developed specifically for use on the APY Lands to assess retail store practices in key areas of food security (rating product availability, placement and promotion) and the number of varieties of vegetables and fruits and proportion of unsweetened drinks to SSBs against targets articulated in the revised Mai Wiru nutrition policy. The FIRST tool is relevant to remote community stores and is not suitable for application in convenience stores or large supermarkets.

From 2014 to 2022 inclusive, food prices were collected in each store using the Food Alliance for Remote Australia Market Basket tool [43] to assess the fortnightly cost of a selected basket of (mostly) healthy items in each store for a family of six, in order to continue the time-series [44]. From 2018, food prices were collected using the updated and more robust Aboriginal and Torres Strait Islander Healthy Diets ASAP (Australian Standardised Affordability and Pricing) methods protocol as detailed elsewhere [45], consistent with international standards [46] (Supplementary File 1). The latter enabled comparison between stores of the cost, cost differential and affordability of healthy and habitual diets (as reported in the most recent national nutrition surveys [47]), and with data collected elsewhere in Australia. Consistent with previous Australian studies, diets were deemed affordable if they cost 30% or less of household disposable income [48].

### Outcome evaluation

The study outcomes were evaluated by change in community diet assessed by the modified store turnover method, which has been validated previously against objective biomedical data in a successful Aboriginal community nutrition project [18, 49]. Electronic bar code sales data from Mai Wiru stores in April 2018, May 2019 and June 2020 were entered into Excel spreadsheets (by ML, EPH, RCT), assessed for face validity (AL), tallied by food product, divided by the number of days in each month and the mean population in each community [50], and analysed using dietary analysis software [51]. The results were compared with ADG recommendations [4] and available community store turnover data from 2012 [33]. The management of the other stores not managed by Mai Wiru did not agree to provide access to store sales data, so estimates of dietary intake are not available for those communities.

### Analysis and missing data

All available data were analysed and compared by group in the concerted intervention implemented from mid-2018 to mid-2019: intervention focus communities/stores (*n *= 2; IMW1 and IMW2); nutrition policy control communities/stores (*n *= 3; PCMW3, PCMW4 and PCMW5); other APY Control communities/stores (*n* = 3; CAPY6, CAPY7 and CAPY8); and for food price data only, a comparison convenience store located close to, but outside, the APY Lands (*n* = 1; CS9) and regional centre comparison supermarkets in Alice Springs (*n* = 3; CAS10, CAS11 and CAS12). As each community is counted as one location and the number of locations is small *(n* = 8 for FIRST surveys assessing product availability, placement and promotion, and *n* = 12 for pricing surveys) quantitative statistical analysis was not warranted; results are described qualitatively.

On six out of 72 occasions, the FIRST survey could not be conducted in stores; for example due to temporary store closure and stocktaking. In those cases, the mean score for availability, product placement and promotion for that store was used to impute relevant data. On four out of 60 occasions the Healthy Diets ASAP survey could not be collected in stores. In those cases, the mean price change of habitual and healthy diets on the APY Lands since the previous survey was used to impute relevant data.

### Impacts of the COVID-19 pandemic on study design

From early 2020, the communities of the APY Lands were closed to many service providers due to public health measures to restrict movement and decrease risk of transmission of COVID-19. Retail store monitoring and surveillance was postponed, NPY Women’s Council nutrition case management services on the APY Lands were suspended, and retail stores were subject to supply and staffing difficulties. All travel by Anangu, including for cultural reasons, was controlled to try to keep people well in their home communities. Electronic withdrawal of cash from savings accounts was also suspended, making it more difficult for those who preferred to use cash for store food purchases.

To offset some of the economic impacts of the pandemic, in 2020 the Australian Government provided additional supplements to recipients of some welfare payments [52]. This likely impacted food affordability on the APY Lands for households in which there were Anangu entitled to the additional income.

From 2021 to 2023, community ‘lockdowns’ continued more sporadically, and some nutrition services and store monitoring and surveillance activities recommenced. One important development in 2021 was the establishment, training and support of the NPY Women’s Council Anangu Research Team to collect data from community retail stores. Information on availability, placement and promotion, price/affordability, and sales/turnover of foods was collected where available.

Due to the COVID-19 pandemic, planned, intensive strategies to tackle food security from 2020 could not be maintained; nevertheless, analysis and synthesis of all available data on the implementation, impacts and outcomes of community-led efforts to improve food security and diet on the APY Lands provides a compelling case study.

## Results

### Strategies to improve food supply and increase demand for healthy foods

Most strategies were implemented as intended from mid-2018 to mid-2019, including release of the revised Mai Wiru nutrition policy developed early in 2018. However, ‘business as usual’ was not possible from early 2020 for the reasons described earlier. Local interest and involvement in the program increased following establishment of the Anangu Research Team.

From mid-2018 to mid-2019 in the two focus communities (IMW1 and IMW2), the community-based project officer received 105 requests for assistance with formal nutrition promotion activities (Table 2). Ninety-three percent of these activities involved *malpas*, remunerated for their work, and/or service providers. The most popular sessions (39%) involved practical cooking. Twice the number of cooking sessions were requested at IMW1 than IMW2, but nearly five times more store-based activities were requested at IMW2. The number of bush excursions and resource development activities were similar at both communities.

**Table 2:** Number of activities, by type, conducted by community-based project officer, *malpas* and service providers in communities IMW1 and IMW2, 2018 to 2019

**Type of activity**		**Community/** **store IMW1**	**Community/** **store IMW2**	**Total**	**Number with support of** *malpas* **and/or service providers**
Community-based activities to increase demand for healthy food and drinks	Bush excursions (bush picnics, bush food trips, and camps)	10	9	19	19
	Cooking at school, youth shed, community development program etc.	26	18	44	42
	Development of nutrition resources including low-cost healthy recipe posters etc.	14	13	27	21
Formal store-based activities to help improve supply of healthy foods	Store-based support for revised nutrition policy in collaboration with store managers and store committees, included cooking workshops at the store, food tastings, store tours etc.	4	19	23	23
	Total	54	59	113	105

### Store food security indicators

**Fig. 3:**
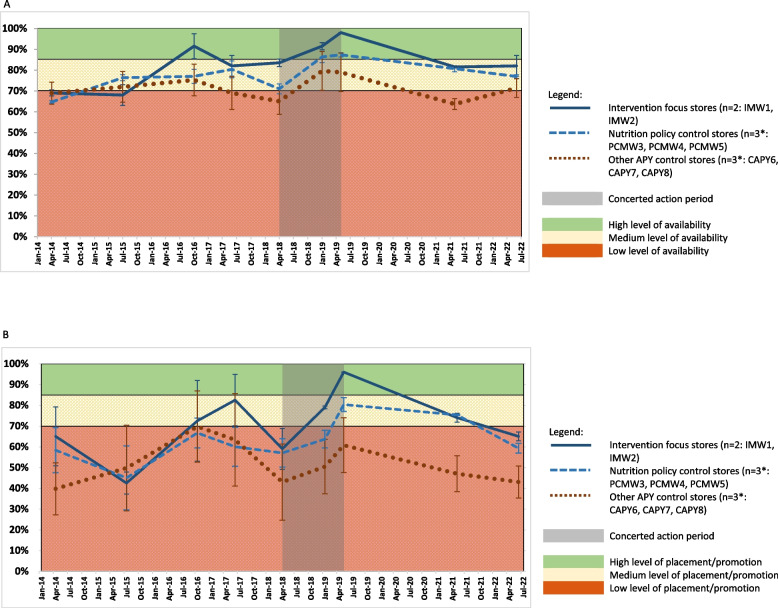
Availability, product placement and promotion of healthy and unhealthy foods and drinks in stores on the APY Lands, 2014 to 2022. **A** Availability of healthy and unhealthy products in stores on the APY Lands, 2014 to 2022 (mean score ± SE). *missing data imputed as described in Methods. **B** Placement and promotion of healthy and unhealthy products in stores on the APY Lands, 2014 to 2022 (mean score ± SE). *missing data imputed as described in Methods

The results of the FIRST surveys of the availability, placement and promotion of healthy and unhealthy foods in the grouped community retail stores from 2014 to 2022 inclusive are presented in Fig. 3. The corresponding detailed FIRST data available for each store are included in Supplementary File 2.

From 2014 to 2018, Mai Wiru stores tended to score similar or slightly higher for availability of healthy and unhealthy foods and drinks than other stores on the APY Lands (Fig. 3A), although variance was high, especially among those stores managed by organisations other than Mai Wiru. From mid-2018 to mid-2019, product availability scores assessed in the two intervention focus stores (IMW1 and IMW2) improved to nearly 100% and with increased velocity compared to other stores where product availability scores increased at similar rates. Product availability scores decreased in all stores after 2019 but remained highest in Mai Wiru stores and particularly in the two intervention focus stores.

From mid-2018 to mid-2019, the number of types of fresh fruit and vegetables stocked in the intervention focus stores (IMW1 and IMW2) increased from 11 to 19 and from 17 to 35 respectively; this was almost double the increase assessed in other stores on the APY Lands over the same period. While the variety of fresh vegetables remained high at 35, the numbers of types of fruit stocked in the intervention focus stores had decreased to 12 by 2022, although these stores still displayed a greater variety of all fresh produce compared to other stores. Also, from mid-2018 to mid-2019, the proportion of wholegrain bread displayed increased by over 20% at the two intervention focus stores, but by less than 5% at the other Mai Wiru stores.

Conversely, the proportion of refrigerator and shelf space stocked with SSBs decreased from mid-2018 to mid-2019 by 46% (from 35 to 19%) in the two intervention focus stores (IMW1 and IMW2) but was relatively stable at higher levels (49% to 46%) in the other Mai Wiru (nutrition policy control) stores, and decreased by only 15% from a high level (around 63%) in other stores on the APY Lands. However, there was a large variance in the latter category with, at the request of the community, one store (CAPYS7) only selling SSBs on Friday from 2019, and no SSBs at all from 2021.

A similar pattern to the overall product availability scores was seen in the scores for product placement and promotion from 2014 to mid-2022 (Fig. 3B); for example, scores for all metrics improved markedly to above 95% in the intervention focus stores (IMW1 and IMW2) from mid-2018 to mid2019. For the following two years, product placement and promotion remained higher in Mai Wiru stores, and especially in the two intervention focus communities, than other stores on the APY Lands.

### Food and diet price and affordability

**Fig. 4:**
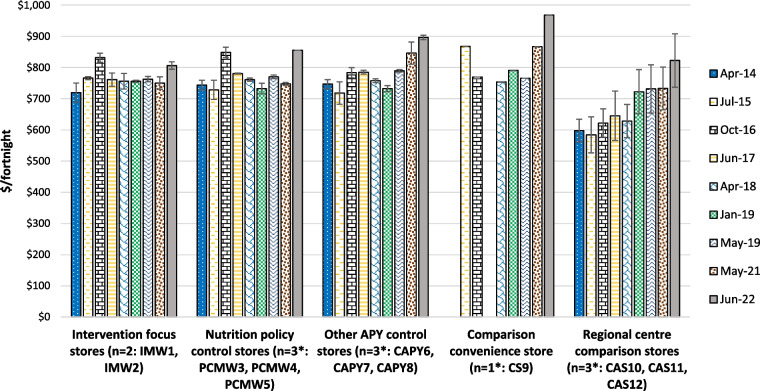
Cost of the FARA Market Basket for a household of six per fortnight in stores on the APY Lands and comparison locations where available, 2014 to 2022 (mean ± SE). * missing data imputed as described in Methods; see Supplementary File 3

The costs of a basket of foods assessed by application of the Food Alliance for Remote Australia (FARA) Market Basket tool in the grouped community retail stores are presented in Fig. 4. Available data for individual stores are included in Supplementary File 3.

The cost of the market basket was at least 20% less expensive in Alice Springs than on the APY Lands. However, prices in the small Alice Springs supermarket that was most popular with Aboriginal customers were more comparable with those on the APY Lands, contributing to the high variance seen in food prices in that regional centre. Except for notable peaks of around 8% increase in Mai Wiru stores in October 2016 and the dramatic increase of around 10% observed in most stores from May 2021 to June 2022, prices of the market basket of foods tended to increase quite moderately over time, especially in Mai Wiru stores.

**Fig. 5:**
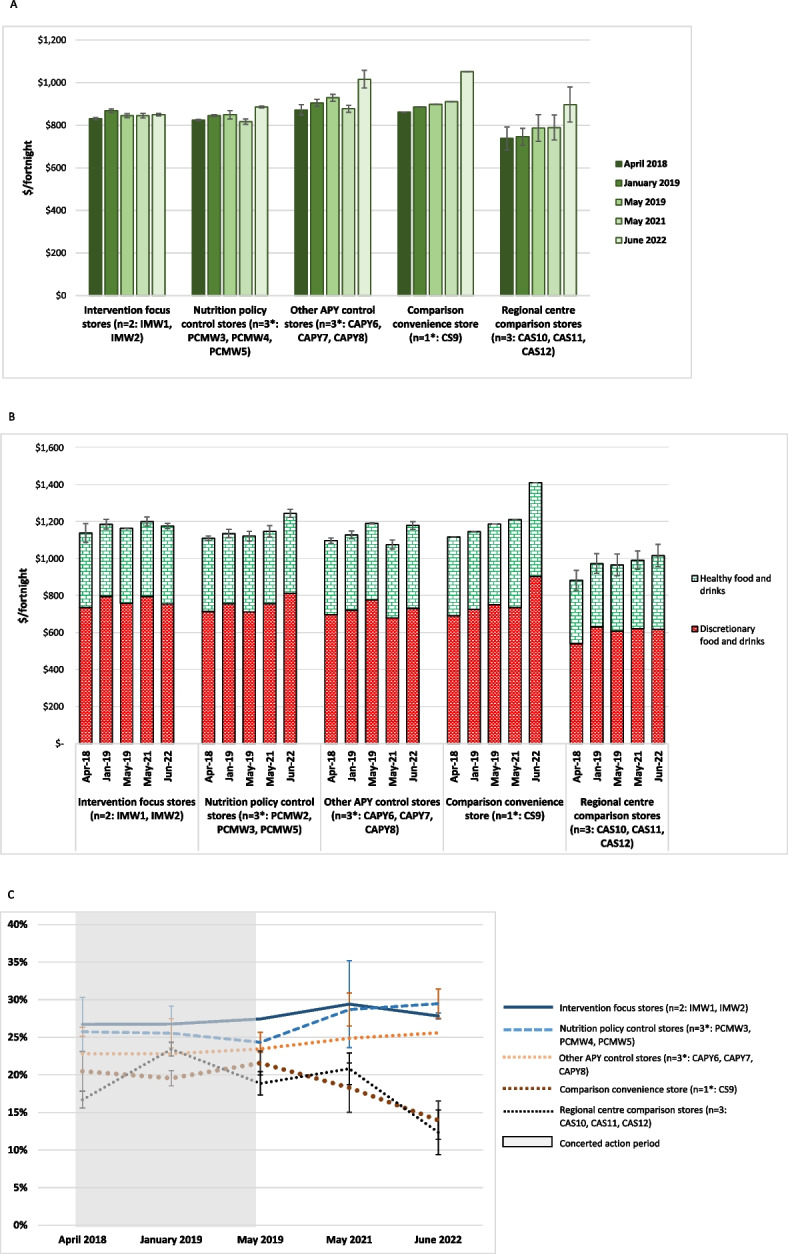
Cost of diets on the APY Lands and comparison locations, 2018–2022 (mean ± SE). **A** Cost of a Healthy Diet for a family of four per fortnight on the APY Lands and comparison locations, 2018 to 2022 (mean ± SE). * missing data imputed as described in Methods. **B** Cost of the Habitual Diet for a family of four per fortnight on the APY Lands and comparison locations, 2018 to 2022 (mean ± SE). * missing data imputed as described in Methods. **C** Differential cost of habitual diet and healthy diets on the APY Lands and comparison locations, 2018 to 2022 (mean ± SE). * missing data imputed as described in Methods

Application of the Aboriginal and Torres Strait Island Healthy Diets ASAP methods protocol from April 2018 showed that healthy diets on the APY Lands cost $820 to $1,050 per fortnight for a household of four people, around 25% more than in Alice Springs (Fig. 5A). Food prices tended to be lower and more stable in Mai Wiru stores than in the convenience store outside the APY Lands or in the other stores on the APY Lands, although there was a large variation in the latter. Compared to other stores on the APY Lands, from mid-2018 the cost of the healthy diet was relatively low in the two intervention focus stores (IMW1 and IMW2). Except in those two stores, there was a marked increase in food prices from May 2021 to June 2022. By 2022, the cost of the healthy diet in the Mai Wiru stores, particularly in the two focus communities, was similar to that in Alice Springs. This was also the case for fruit and vegetables specifically, which in June 2022 comprised 32% of the total cost of healthy diets in Mai Wiru stores and in Alice Springs, but up to 40% in other stores.

### Relative cost of habitual and healthy diets

The cost of the habitual diet assessed by the Aboriginal and Torres Strait Islander Healthy Diets ASAP protocol is presented in Fig. 5B; this also shows the relative proportion of the cost of the habitual diet derived from healthy and unhealthy components. Unhealthy foods and drinks comprised over 60% of the cost of the habitual diet in the communities on the APY Lands. The differential cost of healthy and habitual diets is presented in Fig. 5C. A healthy diet would cost 14% to 30% less than the habitual diet on the APY Lands, with the cost differential and potential cost savings of up to $353 per household per fortnight, highest in Mai Wiru stores. Compared to Mai Wiru stores, the cost differential between habitual and healthy diets decreased markedly from 2021 to 2022 in other stores on the APY Lands and in Alice Springs. (Fig. 5C).

### Affordability of healthy diets

**Fig. 6:**
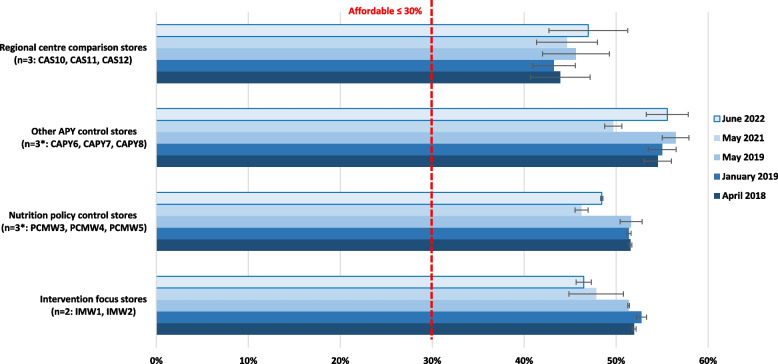
Affordability of healthy diets on the APY Lands and comparison locations, 2018 to 2022 (percentage of welfare dependent household income, mean ± SE). * missing data imputed as described in Methods

The estimated welfare incomes for the reference households are included in Supplementary File 5. On the APY Lands and in Alice Springs respectively per fortnight they ranged from $1,600 and $1,680 in April 2018 to $1,829 and $1,910 in June 2022. At all times assessed, healthy diets were unaffordable on the APY Lands, costing from 46 to 57% of household income for those relying on welfare benefits (Fig. 6). In Alice Springs, at around 45% of household income, healthy diets were also unaffordable for those relying on welfare benefits, although around one third more affordable than on the APY Lands (Fig. 6). As welfare incomes were the same in each community throughout the APY Lands at each time point, patterns of affordability of healthy diets reflected the price of healthy foods in the different community stores. Healthy diets were consistently around 6% more affordable in Mai Wiru stores than other stores on the APY Lands. However, there was a large variation in affordability of healthy diets in non-Mai Wiru stores. In the two intervention focus communities with Mai Wiru stores (IMW1 and IMW2), affordability of the healthy diet improved by 8% from mid-2018 to mid-2019, and by another 2% to mid-2022. Similar results were seen initially in the other communities with Mai Wiru stores, but after 2019 affordability of healthy diets worsened by over 10% in these and all other communities on the APY Lands (Fig. 6).

### Store bar-code sales turnover and apparent community dietary intake

**Fig. 7:**
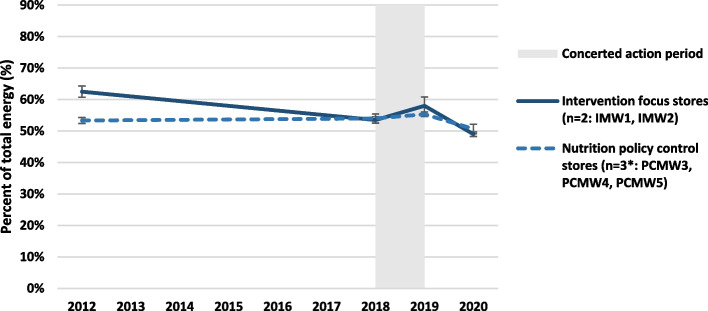
Proportion of energy of food sales derived from healthy foods in Mai Wiru stores (mean ± SE)

Analysis of available bar code sales data showed the proportion of total energy turnover derived from healthy foods in the two focus communities with Mai Wiru stores decreased from 63% in April 2012 to 54% in May 2018 before increasing to 58% in May 2019 (Fig. 7). Also, in IMW1 and IMW2 from mid-2018 to mid-2019, the apparent intake of fresh fruit and vegetables increased from 14.6 to 17.0 g per 1000 kJ, bread decreased from 14.6 to 13.4 g per 1000 kJ (with 25% increase in wholegrain varieties) and SSBs decreased from 39 to 37% of total volume of drinks sold.

In contrast, such improvements were not observed from mid-2018 to mid-2019 in the other communities with Mai Wiru stores, where the proportion of healthy foods remained stable at 54% energy. In these policy control communities, the intake of fresh fruit and vegetables decreased from 19.1 to 15.5 g per 1000 kJ, bread decreased from 14.0 to 12.5 g per 1000 kJ (with 22% increase in wholegrain varieties) and SSBs increased from 59 to 63% of total volume of drinks sold.

However, after 2020 the proportion of healthy foods decreased in all Mai Wiru stores; to 51% in the intervention focus stores and to 49% in the policy control stores. This deterioration in apparent community diet quality was due mainly to increased turnover of unhealthy take-away and convenience foods (from 4 to 11% energy) and increased turnover of SSBs (from 4 to 8% energy). This was offset by 29% decreased turnover of bread and cereals, especially wholegrain varieties (from 24 to 17% energy) and decreased intake of fruit and vegetables to pre-COVID-19 levels.

## Discussion

This study highlights the collective efforts of the NPY Women’s Council, Nganampa Health Council and Mai Wiru regional stores to improve food security and nutrition on the APY Lands over the past decade, building on learnings and local strengths identified previously [33, 36]. In the 12 months from mid-2018, all food security metrics including availability, placement and promotion, and price of healthy foods improved most in the two communities leading focussed store and nutrition promotion activities (IMW1 and IMW2). Importantly, during this time, dietary intake improved in these two communities only. Impacts were less marked in the three communities whose Mai Wiru stores were just exposed to the revised nutrition policy (PCMW3, PCMW4, PCMW5). In the other three communities that experienced business as usual (CAPY6, CAPY7, CAPY8), impacts were even less apparent, but more varied. For example, in response to requests from the local community council who were concerned about health impacts, the availability of SSBs was lowest in CAPY7 at every survey. The availability of SSBs was also relatively low consistently in IMW1; likely this was also due to the request of the local community council for no ‘full strength Coke’ (Coca Cola) to be stocked in the store, which dated back to 2008 [53].

Such long interest of community councils, local store committees and key community leaders in food security and nutrition was reflected in the performance of retail stores in IMW1, IMW2 and CAPY7 prior to 2018 (Fig. 3) [33]. This interest drove the request for additional focus on IMW1 and IMW2 that initiated this study. This was underscored by strong, long-standing personal and familial relationships between Anangu residing in these communities, NPY Women’s Council, Nganampa Health Council and the research team [36]. Feedback of store survey results to community leaders and all service providers remains critical to inform decisions affecting food security policy and practice. In this regard, in 2021 the development of the Anangu Research Team to collect food prices and other food security metrics in stores and to advocate for evidence-informed action based on the results, has been an extremely positive development.

The performance of most stores changed with different store managers; the power and influence of store managers as food security and nutrition ‘gate keepers’ has been long recognised [54]. From mid-2018 to mid-2019 at IMW2 two different store managers were employed; both were keen to make improvements consistent with the revised Mai Wiru store nutrition policy, collaborating actively with cooking demonstrations, food tastings, developing healthy takeaways, nutrition training for store staff, extending store opening hours at the weekend and hosting healthy community events, such as movie nights. Over the same period, IMW1 employed three different store managers and seven temporary management teams. While they were aware of the need to work with the community-based project officer and community members to comply with the revised Mai Wiru store nutrition policy, none was as proactive as those at IMW2. Community members regularly expressed dismay at the high turnover of store management, as much time and effort were spent forming relationships and getting to understand the structures put in place by different individuals. However, some store managers did illustrate what might be possible. For example, in IMW1 for one week in 2018, the store sold over 800 healthy takeaway meals prepared by temporary staff and trainees from the local school. The initiative was discontinued when the staff left the community but demonstrated clearly that Anangu value the availability of healthy, nutritious, convenient options; several community members have since requested training to maintain the program.

Importantly, especially in the face of high turnover of store staff, the store survey results demonstrated the value of a documented and well promoted and supported store nutrition policy. The additional capacity and resources invested in the activities of the community-based project officer/nutritionist and local Anangu *malpas* were also vital to success, as evidenced by the achievements of IMW1 and IMW2 from mid-2018 to mid-2019. The many local, practical in-store and community nutrition activities (Table 2) contributed clearly to the higher scores for promotion, impacts on availability, access and affordability of healthy foods, and improved dietary outcomes, seen in these communities during the concerted intervention.

From early 2020 major disruptions and ‘lockdowns’ related to the COVID-19 pandemic affected service delivery, store operations and data collection, and from this time it was not possible to maintain ‘business as usual’ to tackle food security. By April 2021 when it was possible to collect store data again, there had been a notable decline in all food security metrics. This suggests that regular scrutiny of stores may have contributed to previous improvements. However, the decline was less marked in Mai Wiru stores than others, and among these, food security indicators were most resilient in IMW1 and IMW2.

Impact evaluation also focussed on the cost of and affordability of healthy diets; these are affected by multiple determinants. Longitudinal price data assessed by the FARA Market Basket tool illustrated the value of store monitoring and surveillance; if regular store surveys had not been in place, the 8% increase in Mai Wiru stores in October 2016 would likely have gone undetected. Investigations at the time identified an error in ordering algorithms that was rectified rapidly. Unfortunately, the 10% increase in food prices observed from May 2021 to June 2022 was not so easily fixed. This corresponded to national CPI (food) data, reflecting the impacts of the COVID-19 pandemic, bushfires, floods, and hikes in fuel and energy prices as a result of the war in Ukraine – all factors contributing to a cost-of-living crisis in Australia and globally [55].

However, as the FARA Market Basket tool includes both healthy and unhealthy items, the policy relevance of the results produced are not always clear [44]. For example, if the price of sugar in the basket decreased, contributing to decreased cost of the total basket, this could encourage increased consumption of sugar, which is not a positive outcome from a nutrition perspective. The Aboriginal and Torres Strait Islander Healthy Diets ASAP protocol [45], applied on the APY Lands from 2018, avoids this issue by costing the habitual diet (as reported by participants in the Aboriginal and Torres Strait Islander Nutrition and Physical Activity Survey 2011–13) [3] and also a healthy, equitable, more sustainable diet achieved by minimum change to the habitual diet as recommended in the ADGs [4].

The pattern of results for healthy diet costs and affordability were similar to those of availability, product placement and promotion in that they were more favourable and stable in Mai Wiru stores than other locations on the APY Lands, although there was a high variance in the latter. The relatively high costs of healthy foods in remote areas compared to the regional centre in this study is consistent with previous research findings, which show costs can be up to 50% higher in remote Aboriginal communities [20]. Contributing factors include relative lack of store group buying power; high transport, refrigeration and power costs; and other overheads such as store groups needing to provide housing for store managers in remote areas [20].Therefore, it was remarkable that the cost of healthy diets, and specifically the prices of fresh produce, in the two intervention focus stores (IWM1 and IMW2) were lower than in Alice Springs (CAS10, CAS11 and CAS12) in July 2022. The cost differential between healthy and habitual diets, which can help drive healthier food choices [56], was also highest in the two intervention focus communities with Mai Wiru stores (IMW1 and IMW2) and had increased to nearly 30% by 2022. This is likely due to supported implementation of the revised Mai Wiru nutrition policy, which included strategies to cross-subsidise the price of healthy foods, such as fruit and vegetables which were sold at cost, by increasing the price of unhealthy options. These results contrast markedly with the disproportionate increase in the cost of healthy foods compared to unhealthy foods observed from 2021 to 2022 in Alice Springs and the comparison convenience store (CS9) (Fig. 5B) and elsewhere in Australia [55]. While the cost of a healthy diet was stable in other Mai Wiru stores 9PCMW3, PCMW4, PCM5) from 2019 until 2021, by June 2022 this had increased at nearly double the rate of the habitual diet (up 5.1% compared to 2.7%) and was higher than in stores managed by other groups on the APY Lands (CAPY6, CAPY7, CAPY8). Greater cross-subsidisation of the price of healthy foods in all stores would help address this to reduce cost and improve affordability of healthy diets in all communities on the APY Lands.

Affordability of healthy diets on the APY Lands (46% to 57% of household income for those relying on welfare benefits) was within the range of 34% to 80% of household income estimated in remote Aboriginal communities, which is at least twice that experienced by non-Indigenous households in urban areas [20]. By June 2022, affordability of healthy diets in IMW1 and IMW2 had continued to improve relative to all other locations, but at 46% was still unaffordable (being ≤ 30% of household income). As it is difficult to identify how the price of healthy foods could be decreased further in these two communities, particularly at a time of high food price inflation [55], the persistent low affordability of healthy diets on the APY Lands confirms the findings of the *Maitjara Wangkanyi* study highlighting the critical role poverty plays in these communities and the strength of Anangu resourcefulness and resilience in the face of deprivation [36].

Results of analysis of barcode sales are characteristic generally of suboptimal diets associated with poverty [36] being high in refined grain/cereal foods and/or unhealthy take-away and convenience foods [33, 36]. As the size of the population in each community was difficult to determine, particularly during the ‘lockdowns’ associated with COVID-19, indicators of diet quality as a proportion of total energy turnover were most reliable to reflect apparent community dietary outcomes. For the first time since 1986 [33] the relative proportion of healthy foods contributing to apparent energy intake increased in IMW1 and IMW2 during the concerted intervention from mid-2018 to mid-2019. An increase of two percent may appear modest. However, the proportion of energy intake derived from healthy foods at community level is a resilient, validated indicator [18], which in this study was underscored by concurrent increase in turnover of fruit, vegetables and wholegrain bread, and decreased turnover of SSBs – very positive outcomes of improved food security in IMW1 and IMW2 until 2020. Several other studies of store-based interventions combined with nutrition promotion in remote First Nations communities in Australia and elsewhere have also reported positive outcomes [19, 23]. However, except for the doubling of fruit and vegetable intake seen in one previous study [18] increased intake of healthy foods has been more modest than observed in 12 months in the two intervention focus communities (IMW1 and IMW2)) in the current study.

Worryingly, the deterioration in apparent community diet quality in all stores seen during the COVID-19 pandemic – when the proportion of energy of food sales derived from healthy foods decreased to less than 50% for the first time – was driven by a 29% decrease in turnover of bread and cereals especially wholegrain varieties, replaced with a doubled turnover of both unhealthy take-away/convenience foods and SSBs. Since the mid-1980s, as a coping strategy against food insecurity, the communities of the APY Lands had relied on cereal foods including bread, damper and *argnu* to assuage hunger [31, 33, 36]. However, in 2020, unhealthy take-away/convenience foods played that role.

Several confounders likely affected these outcomes. As mentioned briefly above, in July 2020 in response to the COVID-19 pandemic, the Australian Government provided additional supplementary payments for recipients of some welfare payments, including those without work (JobSeeker), to which many Anangu would have been entitled [52]. Two additional one-off payments were also provided to some welfare recipients, with people receiving JobSeeker being eligible for one of these in April 2020. This would have resulted in an estimated increase in fortnightly welfare payments from about $1,700 in 2019 to around $3,000 (44% more) in mid-2020 for a household of two unemployed adults and two children. The additional COVID-related payments were reduced in September 2020, then reduced again in January 2021, before being phased out at the end of March 2021 [52]. The extent of other changes in community income during these stressful times for families on the APY Lands, for example due to reduced sales of art works, and/or changes in expenditure on non-food items such as the expensive whitegoods for sale in community retail stores, is unknown.

Unfortunately, the period of additional COVID-19-related welfare supplements to Anangu household income from mid-2020 was not aligned to the period of optimal compliance with the revised store nutrition policy that occurred a year earlier. Despite temporarily higher household incomes, the decreased availability, affordability, placement and promotion of healthy options in stores after 2019 likely contributed to less healthy food and drink being purchased in community stores on the APY Lands in 2020. The marked increase of unhealthy take-away/convenience foods as a proportion of total dietary energy turnover through Mai Wiru stores from 2019 to 2020 also suggested that overcrowding and lack of facilities for cooking, food preparation and storage in housing provided for Anangu had not improved. Neither were NPY Women’s Council staff or the community-based project officer available to facilitate practical solutions during ‘lockdowns’. Hence, worsening food security contributed to poor diets, undermining gains achieved previously.

### Limitations

As could be expected from its ‘real-world’ design, there are several limitations in this study. These include that not all data were always available for all stores; for example, some stores declined involvement during stocktaking periods. Only the Mai Wiru store group agreed to provide access to store barcode sales records for analysis. Food and drink prices only were collected from comparison stores not on the APY Lands, as metrics such as availability, product placement and promotion in large regional supermarkets and convenience stores are not relevant for comparison with remote community stores [57]. An attempt was made to limit the effects of inter-observer bias with the same member of the research team applying the same instrument each store survey and working with the same members of the Anangu Research team once established. However, this was not always achieved, particularly during travel restrictions associated with COVID-19. However, analysis, including of store bar code sales data, was performed by the same researchers in 2018, 2019 and 2020. Statistical analysis was limited due to small numbers, as each community/store corresponded to one data point.

A major limitation is that it is not possible to know if the improvements in food security and diet seen in the focus communities until 2019 may have been maintained if the restrictions due to COVID-19 and subsequent food price inflation had not occurred.

### Implications for policy and practice

Firstly, the results confirmed that, despite the complexity of the issues involved, improvements in food security and diet are possible, still, in remote Aboriginal communities [16–19]. Initial impacts and outcomes were similar in magnitude to those achieved previously in successful community-led nutrition projects [16–19, 23]. The study confirms the need for dedicated community-based capacity to support the implementation of strategies to improve food supply and increase demand for healthy foods. Importantly, the results highlight that optimal availability, affordability, placement, and promotion of healthy foods and drinks in retail stores needs to occur *at the same time* as increased household income to ensure healthy diets. They also demonstrate that efforts need to be sustained. As with previous successful nutrition programs, the work was strongly embedded in service delivery but funded as research, raising issues of sustainability that beset most nutrition programs in remote Aboriginal communities [20]. Greater investment in food security is warranted to improve Aboriginal and Torres Strait Islander health, particularly to address the serious consequences of COVID-19 related restrictions on nutrition and likely increase in diet-related NCD [1].

Results also confirm that it is essential that food security and nutrition projects are led and directed by communities, with the backing of trusted community service organisations [20]. It could be argued that the success of this project until 2020 was due to decades of collaborative work on the APY Lands by community members, NPY Women’s Council, Nganampa Health Council and Mai Wiru Regional Stores Council [33].

The lack of a fully operational store nutrition policy leaves communities vulnerable to the commercial determinants of health [33] and the whims and fad food beliefs of individual managers and other service providers [5, 20]. More could be done to publicise and promote evidence-based store nutrition policies to the community councils that own the stores.

## Conclusions

This project demonstrated that concerted community-lead intervention addressing both supply and demand factors positively impacted the availability, placement and promotion of healthy and unhealthy foods in stores, improved diet cost and affordability, and importantly, improved dietary outcomes in the two focus communities. Relative to other locations, these improvements were maintained during the early months of the COVID-19 pandemic, but gains in food security and diet eroded as the pandemic continued. There is a need for regular, comprehensive, co-ordinated national monitoring of food security in Australia, including in Aboriginal and Torres Strait Islander communities. The study highlights that sustained investment to address food security and nutrition is required urgently in remote First Nations communities. The study informs current gaps in policy and practice to reduce the high rates of diet-related non-communicable disease in First Nations communities in Australia.

## Supplementary Information

Supplementary Material 1.

## Data Availability

The datasets used and/or analysed during the current study are available from the corresponding author on reasonable request.

## Abbreviations

ADGs: Australian Dietary Guidelines
APY: Anangu Pitjantjatjara Yankunytjatjara
ASAP: Australian Standardised Affordability and Pricing
CDP: Community Development Programme
COVID-19: The disease caused by the SARS-CoV-2 virus
FARA: Food Alliance for Remote Australia
FIRST: Food Index for Remote Stores
HAAC: Home and Aged Care
HREC: Human Research Ethics Committees
NATSIHS: National Australian Aboriginal and Torres Strait Islander Health Survey
NCDs: Non-communicable diseases
NPY: Ngaanyatjarra Pitjantjatjara Yankunytjatjara
NPYWC: Ngaanyatjarra Pitjantjatjara Yankunytjatjara Women’s Council
RASAC: Regional Anangu Services Aboriginal Corporation
SSBs: Sugar sweetened beverages
UPK: Uwankara Palyanyku Kanyintjaku

## Footnotes

1 Mai Wiru means “good food” in the Pitjantjatjara language.

2 Partnering non-Indigenous workers with an Anangu “malpa”, a cultural broker/colleague/translator.
