# Supplementary material for: Making it on the breadline – improving food security on the Anangu Pitjantjatjara Yankunytjatjara Lands, Central Australia
Source: BMC Public Health. 2024 Nov 8;24:3087. doi: 10.1186/s12889-024-20495-9 (PMC11545495; doi:10.1186/s12889-024-20495-9)
Supplement: Supplementary file 1 — Supplementary Material 1. [file 12889_2024_20495_MOESM1_ESM.docx]

**Additional files**

Additional file 1: Survey instruments and tools.

Additional file 2: Availability, placement and promotion of foods and drinks in individual retail stores on the APY Lands.

Additional file 3: Cost of FARA Market Basket in individual retail stores on the APY Lands.

Additional file 4: Cost of habitual and healthy diets for a family of four per fortnight in individual retail stores on the APY Lands and comparison locations.

Additional file 5: Welfare incomes for a family of four per fortnight on the APY Lands and comparison locations

**Additional files.**

**Additional File 1. Survey instruments and tools**

1. **FARA Market Basket tool**


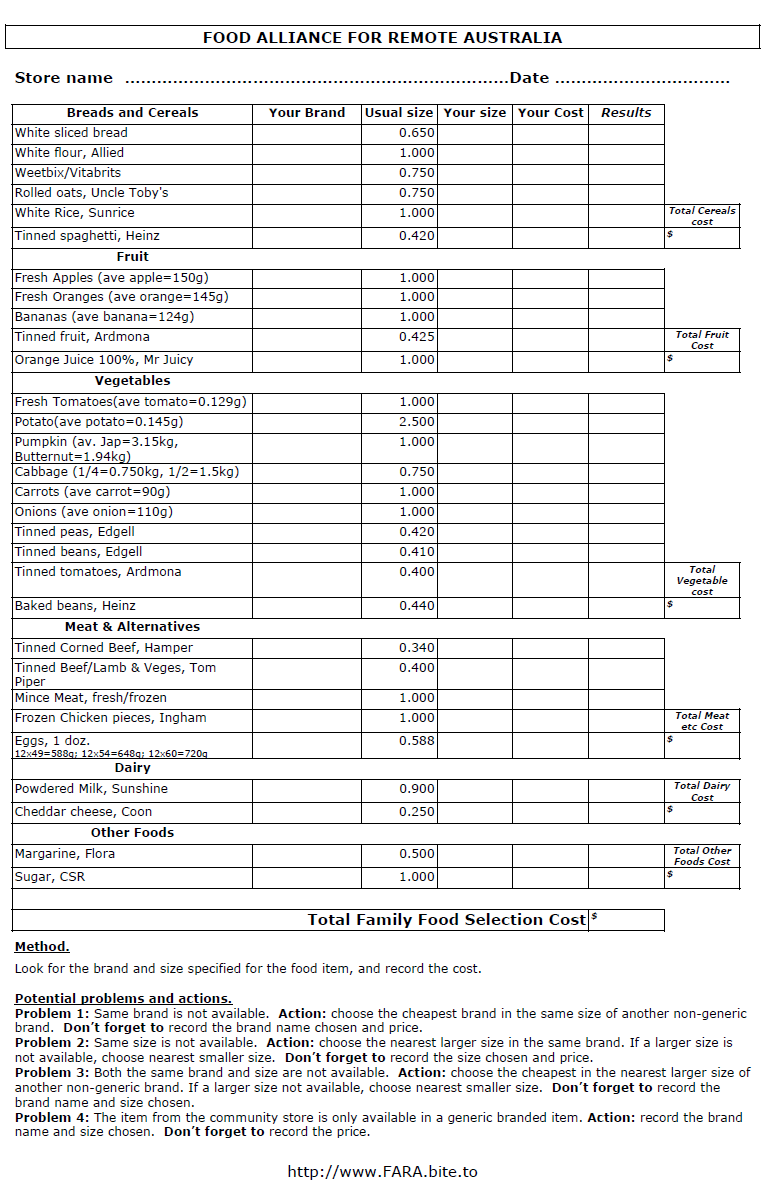


**1B: Components of the FIRST survey tool**


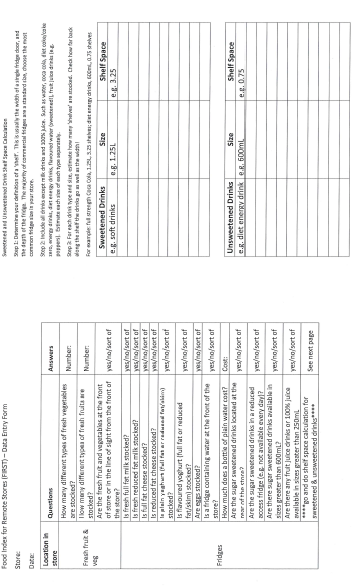

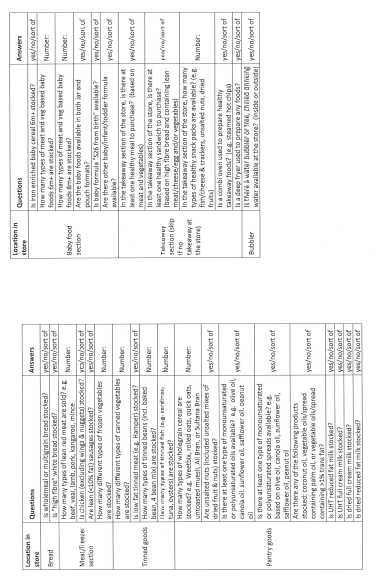

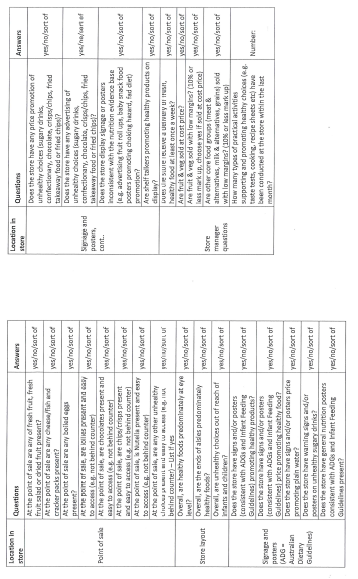


**1C: Aboriginal and Torres Strait Islander Healthy Diets ASAP protocol tool**


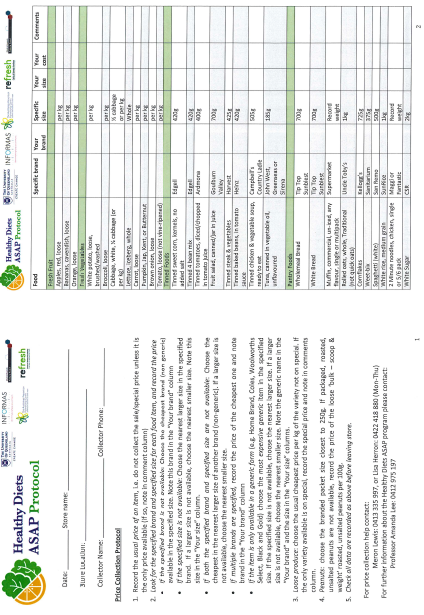

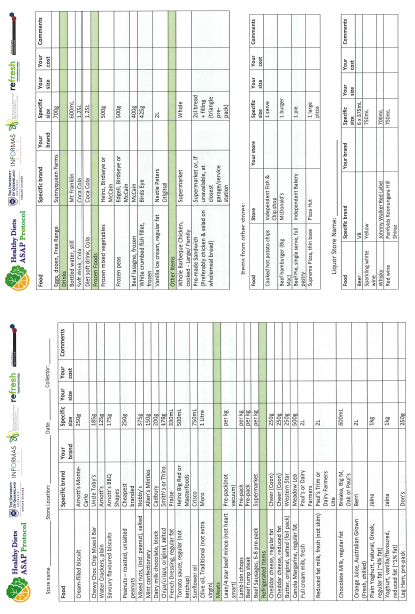


**Additional File 2: Availability, placement and promotion of healthy and unhealthy foods in individual retail stores on the APY Lands.**

**Additional File 2A: Category of availability score of foods and drinks in individual retail stores on the APY Lands, 2014 to 2022**



|  | **Mai Wiru stores on APY Lands** | | | | | **Other stores on APY Lands** | | |
| --- | --- | --- | --- | --- | --- | --- | --- | --- |
|  | **Intervention focus communities** | | **Store nutrition policy control communities** | | | **APY control communities** | | |
|  | **IMW1** | **IMW2** | **PCMW3** | **PCMW4** | **PCMW5** | **CAPYS6** | **CAPYS7** | **CAPYS8** |
| April 2014 | 71 | 67 | 64 | 66 | 64 | 71 | 79 | 57** |
| July 2015 | 61 | 75 | 79 | 73 | 77 | 71 | 88 | 57** |
| Oct 2016 | 83 | 100 | 83 | 69 | 79 | 83 | 86 | 57** |
| June 2017 | 75 | 89 | 82 | 71 | 88 | 75 | 82 | 50 |
| April 2018 | 86 | 81 | 71 | 66 | 76 | 70 | 75 | 50 |
| January 2019 | 94 | 89 | 91 | 80 | 88 | 90 | 92 | 57** |
| May 2019 | 98 | 98 | 88 | 89 | 85 | 94 | 86 | 57** |
| May 2021 | 81 | 82 | 80 | 84 | 78 | 62 | 70 | 59 |
| June 2022 | 89 | 75 | 78 | 75** | 78 | 64 | 82 | 68 |

* prior to 2018 data were analysed against the RIST checklist; results have been moderated against the results of the FIRST checklist used from 2018

** missing data imputed as described in Methods

| **Colour coding category** |
| --- |
| Green = Good (86% to 100%) |
| Amber = Could be improved (70% to 85%) |
| Red = Poor (<70%) |

**Additional File 2B: Category of product placement and promotion score of food and drinks in individual retail stores on the APY Lands, 2014 to 2022**



|  | **Mai Wiru stores on APY Lands** | | | | | **Other stores on APY Lands** | | |
| --- | --- | --- | --- | --- | --- | --- | --- | --- |
|  | **Intervention focus** **communities** | | **Store nutrition policy control communities** | | | **APY control communities** | | |
|  | **IMW1** | **IMW2** | **PCMW3** | **PCMW4** | **PCMW5** | **CAPYS6** | **CAPYS7** | **CAPYS8** |
| April 2014 | 85 | 45 | 45 | 85 | 45 | 20 | 70 | 29** |
| July 2015 | 35 | 50 | 75 | 50 | 10 | 20 | 100 | 29** |
| Oct 2016 | 45 | 100 | 80 | 70 | 50 | 80 | 100 | 29** |
| Jun 2017 | 65 | 100 | 80 | 60 | 40 | 80 | 100 | 10 |
| April 2018 | 45 | 73 | 71 | 42 | 58 | 33 | 86 | 10 |
| January 2019 | 78 | 80 | 73 | 55 | 63 | 40 | 82 | 29** |
| May 2019 | 96 | 96 | 79 | 74 | 88 | 70 | 83 | 29** |
| May 2021 | 71 | 77 | 75 | 75 | 76 | 34 | 39 | 68 |
| June 2022 | 68 | 62 | 60 | 64* | 54 | 39 | 61 | 29 |

* prior to 2018 data were analysed against the RIST checklist; results have been moderated against the results of the FIRST checklist used from 2018

** missing data imputed as described in Methods

| Colour coding category |
| --- |
| Green = Good (86% to 100%) |
| Amber = Could be improved (70% to 85%) |
| Red = Poor (<70%) |

**Additional File 3: Cost of FARA Market Basket in individual retail stores on the APY Lands**

**Additional File 4: Cost of habitual and healthy diets for a family of four per fortnight in individual retail stores on the APY Lands and comparison locations**

|  | **IMW1** | | **IMW2** | | **PCMW3** | | **PCMW4** | | **PCMW5** | | **CAPYS6** | | **CAPYS7** | | **CAPYS8** | | **CS9** | | **Regional centre comparison stores (mean ± std. error)** | | |
| --- | --- | --- | --- | --- | --- | --- | --- | --- | --- | --- | --- | --- | --- | --- | --- | --- | --- | --- | --- | --- | --- |
|  | Habitual diet | Healthy diet | Habitual diet | Healthy diet | Habitual diet | Healthy diet | Habitual diet | Healthy diet | Habitual diet | Healthy diet | Habitual diet | Healthy diet | Habitual diet | Healthy diet | Habitual diet | Healthy diet | Habitual diet | Healthy diet | Habitual diet | Healthy diet |  |
| Apr 2018 | $1067 | $836 | $1212 | $827 | $1091 | $822 | $1101 | $822 | $1141 | $831 | $1070 | $915 | $1095 | $817 | $1129 | $886 | $1118 | $862 | $832 ± 54 | $683 ± 54 |  |
| Jan 2019 | $1150 | $880 | $1224 | $855 | $1080 | $855 | $1165 | $842 | $1165 | $839 | $1134 | $938 | $1082 | $867 | $1158 | $910 | $1147 | $886 | $918 ± 54 | $710 ± 40 |  |
| May 2019 | $1167 | $847 | $1162 | $842 | $1060 | $821 | $1164 | $897 | $1146 | $830 | $1186 | $970 | $1203 | $903 | $1171 | $915 | $1174 | $898 | $905 ± 59 | $718 ± 63 |  |
| May 2021 | $1235 | $771 | $1167 | $919 | $1087 | $801 | $1216 | $802 | $1142 | $848 | $1089 | $847 | $1021 | $916 | $1122 | $870 | $1213 | $911 | $949 ± 49 | $739 ± 59 |  |
| Jun 2022 | $1198 | $872 | $1157 | $829 | $1201 | $888 | $1274 | $876 | $1247 | $894 | $1138 | $988 | $1178 | $946 | $1226 | $1116 | $1413 | $1051 | $958 ± 60 | $807 ± 82 |  |

**Additional File 5. Welfare incomes for a family of four per fortnight on the APY Lands and comparison locations**

| **Welfare Income** | **APY Lands** | **Alice Springs** |
| --- | --- | --- |
| **April 2018** | $1,600.00 | $1,680.00 |
| **January 2019** | $1,645.14 | $1,725.71 |
| **May 2019** | $1,645.14 | $1,725.71 |
| **May 2021** | $1,766.68 | $1,766.68 |
| **June 2022** | $1,829.45 | $1,909.95 |
